# Input data for mathematical modeling and numerical simulation of switched reluctance machines

**DOI:** 10.1016/j.dib.2017.07.044

**Published:** 2017-07-20

**Authors:** Ali Asghar Memon, Muhammad Mujtaba Shaikh

**Affiliations:** a Department of Electrical Engineering, Mehran University of Engineering and Technology, Jamshoro, Pakistan; b Department of Basic Sciences and Related Studies, Mehran University of Engineering and Technology, Jamshoro, Pakistan

**Keywords:** Switched reluctance machine, Flux linkage, Static torque, Lab VIEW

## Abstract

The modeling and simulation of Switched Reluctance (SR) machine and drives is challenging for its dual pole salient structure and magnetic saturation. This paper presents the input data in form of experimentally obtained magnetization characteristics. This data was used for computer simulation based model of SR machine, “Selecting Best Interpolation Technique for Simulation Modeling of Switched Reluctance Machine” [1], “Modeling of Static Characteristics of Switched Reluctance Motor” [2]. This data is primary source of other data tables of co energy and static torque which are also among the required data essential for the simulation and can be derived from this data. The procedure and experimental setup for collection of the data is presented in detail.

**Specifications Table**

**Table:** 

Subject area	Energy Efficient Motors, Electronic and Electrical Engineering, and Drives, Numerical simulation and modeling of SR machines
More specific subject area	Switched Reluctance Machines
Type of data Tables	Figures, Tables
How data was acquired	Through experimental rig on existing prototype (4phase, 8/6 pole rotary Switched Reluctance Machine D-80, 0.75 kW, 1500 rpm, Bifilar wound type)
Data format	Raw, filtered, analyzed
Experimental factors	Selectivity of software used, and reliability and accuracy of instruments used
Experimental features	NA
Data source location	School of Electronic and Electrical Engineering, University of Leeds, United Kingdom
Data accessibility	Data is available with the article

**Value of the Data**

• Flux data is useful for determination of steady state and transient performance of machines
• The presented data is obvious for switched reluctance modeling irrespective of how it is obtained i.e. through experiments or computer softwares.
• This data shows evidence of nonlinear characteristics of the machine.
• Once having this data, remaining data tables are obtained easily and it is easier to find missing data points by interpolation techniques.
• Accurate simulation in different areas relating SR machine is possible with this data if current and torque of the machine counted.

## 1 Data

The data of flux linkage presented in this article is useful for modeling and simulation of existing SR machine especially when phase current, and phase instantaneous torque profile of SR machine are discussed. Furthermore; an ample picture of this data in 3-D is given for purpose of illustration.

## 2 Experimental design, material and methods

### 2.1 Accumulating data of flux linkage characteristics through the experiments

Electrical machines are judged from its electrical and more importantly the magnetic circuit. The data of flux linkage presented in this paper actually epitomizes the magnetic behavior of the existing machine (4phase, 8/6 pole rotary Switched Reluctance Machine D-80, 0.75 kW, 1500 rpm, Bifilar wound type) [1], [2]. There is range of options to get this data e.g. by help of computer software [3], [4], [5], [6], [7], [8], [9], experimentally [9], [10] and by experiments with association of computer software on existing machine [8], [11]. Initial work reported on the measured technique of this data by search coil method and integrator circuit method dated back when switched reluctance machine was in emergent stage. The behavior of magnetic circuit such as fringing flux and magnetic saturation are the most nearest reasons for the trend of the data [12].

**Fig. 1:**
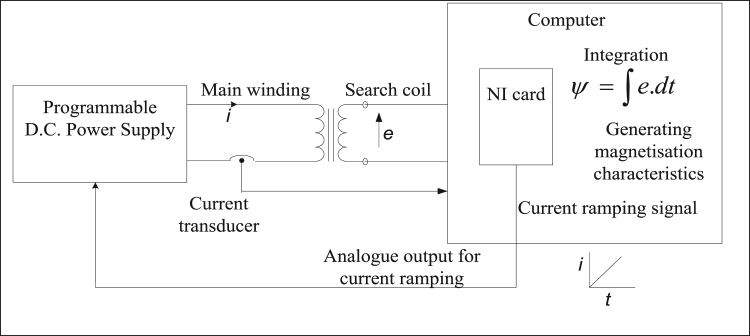
Arrangement for the experiment to get data of flux for different rotor position when different amount of current is allowed to flow through machine.

The experiment setup is shown in Fig. 1 [11], whereas the operating conditions are described in Table 1. This arrangement uses machine with bifilar winding of the machine i.e. search coil to induce EMF (Electro Motive Force), which is then integrated to get data of flux linkage for range of rotor position and current values. The Lab VIEW (Laboratory Virtual Instrumentation Engineering Workbench) software is prioritized for its graphical display and data acquisition.

**Table 1:** Operating conditions.

**Phase current**	**Rotor position**	**Signal produced Time**	**Sampling frequency**	**LabVIEW card**
0 -14 [A]	-30-30 [deg.]	Ramp signal	1 kHz	NI USB 6008

**Fig. 2:**
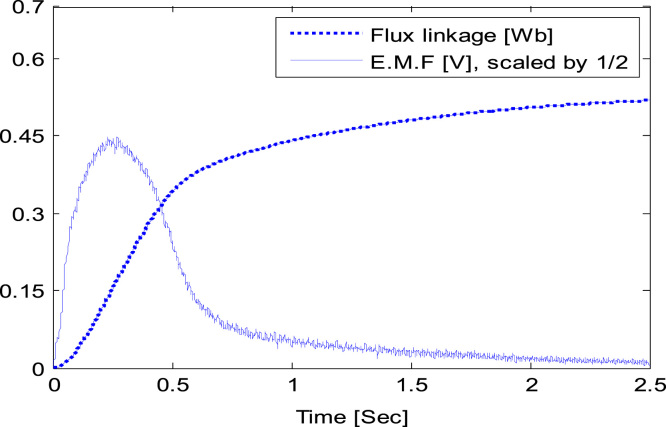
Experimentally obtained induced EMF and flux at aligned position [12].

The obtained data of induced EMF and data of flux after integration is shown in graphical form in Fig. 2.

**Fig. 3:**
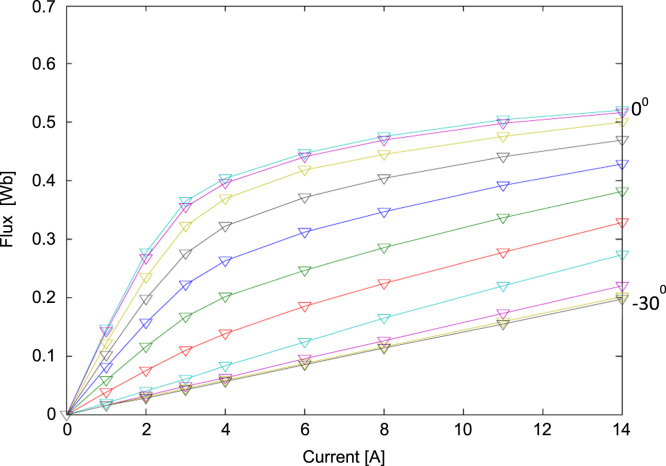
Graphical presentation of measured data of flux linkage data Ψ(θ,i) for different rotor position and current [12].

**Fig. 4:**
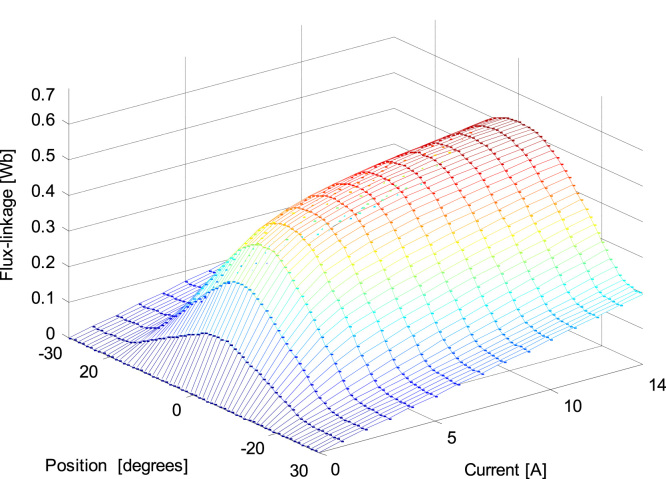
3-Dimensional representation of flux linkage [12].

Measured data of flux for different current values and different rotor position is graphically represented in Fig. 3. The rotor positions marked -30° indicates unaligned position of stator and rotor poles where inductance is minimum, and 0° relate to aligned position of two poles where inductance is maximum. More expounded flux, current and rotor position is shown in Fig. 4. Table 2 shows measured data at two extremes, aligned and unaligned position.

**Table 2:** Measured values of data of flux at two extremes, aligned (0°) and unaligned (-30°) rotor position [12].

Current [A]	0	1	3	4	11	14
-30°	0	0.0144	0.0428	0.0567	0.1552	0.1968
0°	0	0.1461	0.3645	0.4038	0.5039	0.5207

**Fig. 5:**
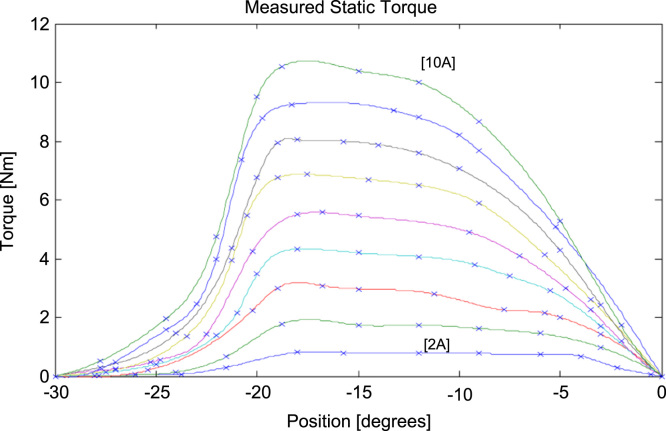
Measured data of static torque [12].

Additional data of static torque is also experimentally obtained by locked rotor test by allowing current to flow through motor winding and measuring toque on dynamometer. The set of measured data in graphical representation can be seen from Fig. 5. Few points of measured data values are shown in Table 3.

**Table 3:** Measured values of data of static torque [12].

Rotor position [deg]	Torque [Nm]	Rotor position [deg]	Torque [Nm]	Rotor position [deg]	Torque [Nm]	Rotor position [deg]	Torque [Nm]
2A	2A	5A	5A	6A	6A	7A	7A
0	0	0	0	0	0	0	0
-9	0.77	-12	4.07	-9.5	4.9	-9	5.87
-30	0	-30	0	-30	0	-30	0
