# Supplementary material for: Input data for mathematical modeling and numerical simulation of switched reluctance machines
Source: Data Brief. 2017 Jul 20;14:138–42. doi: 10.1016/j.dib.2017.07.044 (PMC5567386; doi:10.1016/j.dib.2017.07.044)
Supplement: Supplementary file 1 — Supplementary material [file mmc1.docx]

**CONFLICT OF INTEREST STATEMENT**

**Manuscript No.: DIB-D-17-00483**

**Title: Input data for mathematical modeling and numerical simulation of switched reluctance machines**

We, the authors of this data article, state that we don’t have any conflict of interest with any researcher, editorial member, reviewer, organization, etc.

Regards,

Muhammad Mujtaba Shaikh (corresponding author)

Email: [mujtaba.shaikh@faculty.muet.edu.pk](mailto:mujtaba.shaikh@faculty.muet.edu.pk)

Cell # +92-333-2617602
